# Strand directionality affects cation binding and movement within tetramolecular G-quadruplexes

**DOI:** 10.1093/nar/gks851

**Published:** 2012-09-12

**Authors:** Primož Šket, Antonella Virgilio, Veronica Esposito, Aldo Galeone, Janez Plavec

**Affiliations:** ^1^Slovenian NMR Center, National Institute of Chemistry, Hajdrihova 19, Ljubljana SI-1000, Slovenia, ^2^EN-FIST Center of Excellence, Dunajska 156, Ljubljana SI-1000, Slovenia, ^3^Dipartimento di Chimica delle Sostanze Naturali, Università degli Studi di Napoli Federico II, via D. Montesano 49, Napoli 80131, Italy and ^4^Faculty of Chemistry and Chemical Technology, University of Ljubljana, Askerceva cesta 5, Ljubljana SI-1000, Slovenia

## Abstract

Nuclear magnetic resonance study of G-quadruplex structures formed by d(TG_3_T) and its modified analogs containing a 5′-5′ or 3′-3′ inversion of polarity sites, namely d(3′TG5′-5′G_2_T3′), d(3′T5′-5′G_3_T3′) and d(5′TG3′-3′G_2_T5’) demonstrates formation of G-quadruplex structures with tetrameric topology and distinct cation-binding preferences. All oligonucleotides are able to form quadruplex structures with two binding sites, although the modified oligonucleotides also form, in variable amounts, quadruplex structures with only one bound cation. The inter-quartet cavities at the inversion of polarity sites bind ammonium ions less tightly than a naturally occurring 5′-3′ backbone. Exchange of ^15^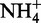 ions between G-quadruplex and bulk solution is faster at the 3′-end in comparison to the 5′-end. In addition to strand directionality, cation movement is influenced by formation of an all-*syn* G-quartet. Formation of such quartet has been observed also for the parent d(TG_3_T) that besides the canonical quadruplex with only all-*anti* G-quartets, forms a tetramolecular parallel quadruplex containing one all-*syn* G-quartet, never observed before in unmodified quadruplex structures.

## INTRODUCTION

Nucleic acid sequences containing short tracts of guanine residues are prone to fold into G-quadruplex structures composed of stacking G-quartets that are described as planar arrangements of four guanines held together by eight hydrogen bonds and monovalent cations (1–4). Sequences that exhibit potential to form G-quadruplex structures are widespread in human genome and they seem to play a role in a number of processes, such as replication, recombination, transcription and translation (5,6). Furthermore, G-quadruplexes form scaffolds of several aptamers, potentially useful as therapeutic agents due to their high affinity and selectivity (7). Aptamers can be obtained by a procedure called SELEX (8) that is a combinatorial chemistry methodology based on oligonucleotide libraries, which are screened for high-affinity binding to a given target. To improve their properties, aptamers are often subjected to post-SELEX modifications involving the base moiety and/or the sugar-phosphate backbone. Among these, the introduction of inversion of polarity sites represents a very useful and chemically accessible post-SELEX backbone modification. As a matter of fact, a 3′-3′ inversion of polarity site has been introduced in the first aptamer approved by the US FDA, namely Macugen, to increase the resistance to the ubiquitous exonucleases (9). Recently, the quadruplex forming thrombin-binding aptamer has been modified by introducing a 5′-5′ inversion of polarity site in a lateral loop, in an attempt to improve its biological activity (10,11). On the other hand, the introduction of inversion of polarity sites inside or at one end of the G-run in tetramolecular quadruplexes, not only does not hinder the structure assembly but it can endow the resulting complex by interesting structural properties, for example, inducing the formation of all-*syn* or *anti-syn-anti-syn* quartets or, in some cases, increasing its thermal stability (12–16). It is well known that quadruplex structures exhibit a remarkable dependency on cations due to the presence of four carbonyl oxygen atoms in the middle of each G-quartet plane (17). Particularly, cations are involved in regulating the structural aspects, the stability and the folding process of quadruplex structures which, from this point of view, are unique among the nucleic acids secondary structures in their metal ions requirements (17). In general, cations can be found between two G-quartets or in the plane of a G-quartet along the central cavity of a G-quadruplex. However, different stacking properties of G-quartets and other structural features make cation-binding sites unequal (18–20). As a consequence, cation-binding sites can be occupied by cations or, alternatively, they can be temporarily vacant or occupied by water molecules (21).

^15^N-labeled ammonium ion as a non-metallic substitute in combination with 2D nuclear magnetic resonance (NMR) spectroscopy represents a unique tool to localize cations and determine their populations at individual binding sites (22–24). Moreover, because cations are not coordinated to their preferred binding sites in a static manner, but they are rather moving between binding sites and bulk solution, NMR also enables to study kinetics of their exchange. Kinetics of cation movement is intrinsically correlated with structural details and local plasticity of specific G-quadruplex topology (25). Our recent studies have demonstrated that kinetics of ^15^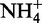 ion movement from the interior of the G-quadruplex core to bulk solution and back is controlled by the topology of loop orientations and specific interactions of loop residues (18,23,24,26,27).

To get deeper insight into the intricate relationships among stability, structural preferences along sugar-phosphate backbone, cation occupancy of binding sites and kinetics of cation movement, we have undertaken a study on G-quadruplex structures containing inversion of polarity sites by utilizing the ^15^N-labeled ammonium ions and NMR spectroscopy. The study involved three 2′-deoxyribo-oligonucleotides [ODNs, d(3′TG5′-5′G_2_T3′), d(3′T5′-5′G_3_T3′) and d(5′TG3′-3′G_2_T5′)] with inversion of polarity sites whose features were compared with the parent quadruplex forming sequence d(TG_3_T). The 3′-3′ or 5′-5′ inversion of polarity sites represent unique backbone modification, because in principle, the size and affinity of an usual cation-binding site defined by the eight guanine carbonyl oxygen atoms between two stacked G-quartets linked by the 5′-3′ canonical sugar-phosphate backbone could be fine tuned, thus providing the cation binding within the structure with desired properties. Particularly, investigations on d(3′TG5′-5′G_2_T3′) and d(5′TG3′-3′G_2_T5′) allowed a direct comparison between the behavior of the binding sites involving natural 5′-3′ and modified 3′-3′ or 5′-5′ sugar-phosphate backbones. In addition, we herein explore how variations in strand directionalities relate structural heterogeneity of G-quadruplexes formed in solution with cation-binding site occupancy and dynamics of cation movement. Furthermore, in contrast to expectations that unmodified oligonucleotides with a single G-tract are supposed to form a standard G-quadruplex structure composed of four parallel strands with only *anti* residues, the data collected for [d(TG_3_T)]_4_ G-quadruplex in this NMR study demonstrates the co-existence of different forms in solution.

## MATERIALS AND METHODS

### Sample preparation

Oligonucleotide d(TG_3_T) was purchased from IDT (Leuven, Belgium). Sample was dissolved in 1 ml of H_2_O and dialyzed extensively against 10 mM LiCl solution. Oligonucleotides with inversion of polarity sites were prepared as described previously (15). The concentrations of the 300 μl NMR samples were between 4.5 and 5.5 mM in single strand. ^15^NH_4_Cl was titrated into the samples with pH between 4.5 and 6.0 to final concentrations of 10 and 80 mM. We have observed only minor spectral changes as a function of pH variation that could be ascribed to the protection of amino groups from exchange with bulk water at lower pH values.

### NMR experiments

All NMR spectra were collected on Varian VNMRS 600 and 800 MHz NMR spectrometers. Experiments were performed at 0 and 25°C. Standard 1D ^1^H spectra were acquired with the use of DPFGSE solvent suppression sequence (28). The 2D NOESY spectra in H_2_O were acquired at mixing times of 80 and 150 ms. Number of ^15^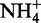-ion-binding sites was determined with the use of ^15^N-^1^H HSQC spectra. ^15^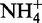 ion movement was followed by a series of ^15^N-^1^H NzExHSQC spectra (22,29,30) at mixing times (*τ*_m_) ranging from 13 ms to 3 s. A binding site is annotated with a single letter code. In the case of isochronous cross-peaks that correspond to several binding sites, a ‘/’ sign has been used (e.g. I2/I2* in Figure 2). The labels of cross-peaks in ^15^N-^1^H NzExHSQC spectra consist of two parts, where the part in square brackets indicates binding site/s within G-quadruplex. The first part of label indicates the initial and the second part the final location of particular ^15^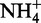 ion over the course of an NMR pulse sequence (e.g. B[I2/I2*] in Figure 7a).

### Data analysis

Volumes of cross-peaks in ^15^N-^1^H HSQC and NzExHSQC spectra were integrated with Varian VNMRJ 2.1B software. Iterative least squares fitting of cross-peak intensities in NzExHSQC spectra versus mixing time (*τ*_m_) was done with Origin 7.5 software (www.originlab.com). Determination of exchange rate constants for ^15^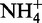 ion movements was done with the use of the following equation: *V*_c_ = *A*[*e − *(*τ*_m_/*T*_1c_)(1 − *e*(*k_N_τ*_m_))], where *V*_c_ represents the volume integral of a cross-peak, *T*_1c_ is the corresponding spin-lattice relaxation time, *τ*_m_ is the mixing time of the 2D NzExHSQC experiment and *k_N_* corresponds to the rate constant for ^15^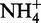 ion movement (23).

## RESULTS

### Inversion of polarity sites drives G-quadruplex formation

**Figure 1.**
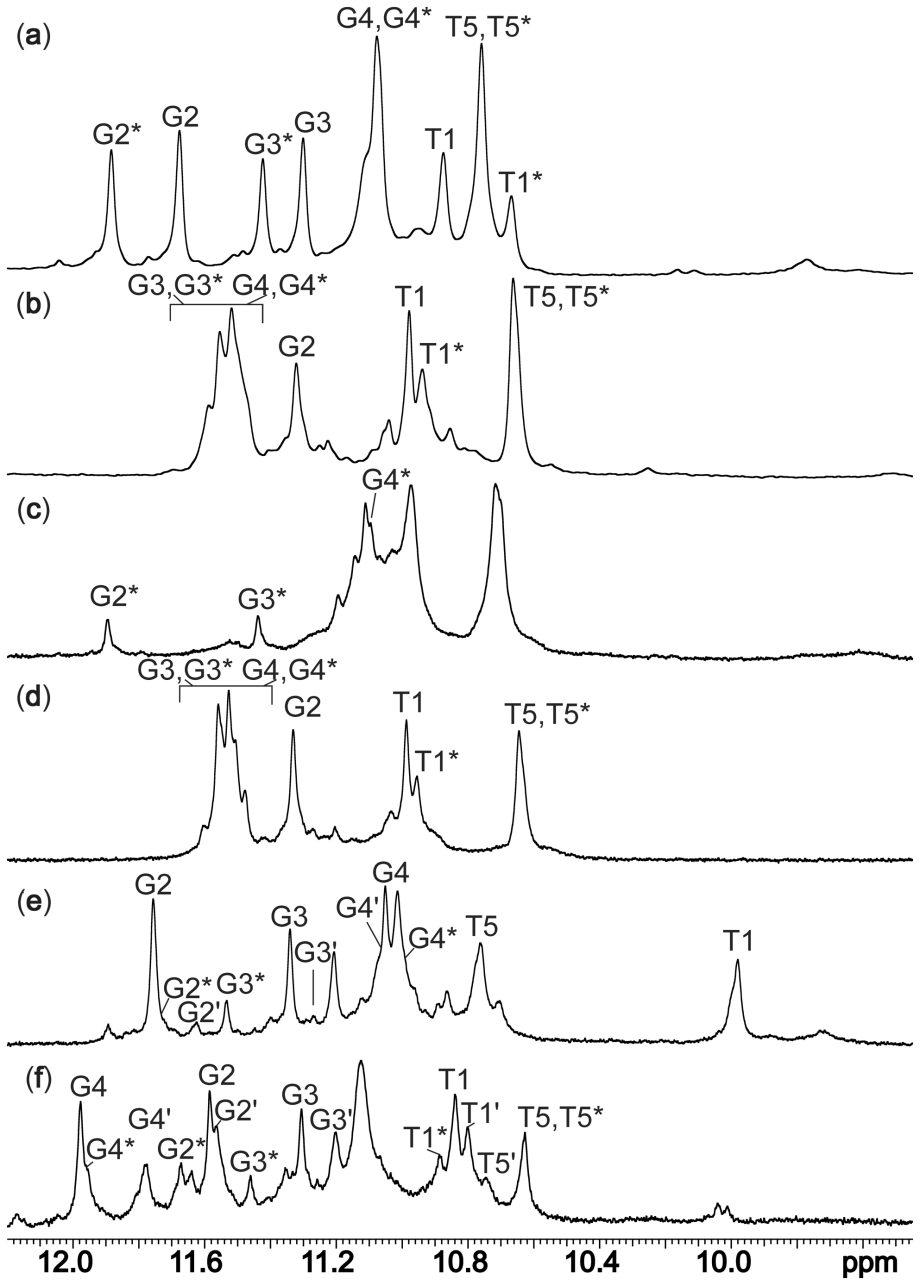
Imino regions of ^1^H NMR spectra of G-quadruplexes formed by d(TG_3_T) (**a**, **c**), d(3′TG5′-5′G_2_T3′) (**b**, **d**), d(3′T5′-5′G_3_T3′) (**e**) and d(5′TG3′-3′G_2_T5′) (**f**) at 0°C in 10% ^2^H_2_O. Concentration of ^15^NH_4_Cl was 10 mM in (a, b) and 80 mM in (c–f). Imino signals of the minor G-quadruplex forms are marked with superscripts (* and ′).

Three oligonucleotides with inversion of polarity sites, d(3′TG5′-5′G_2_T3′), d(3′T5′-5′G_3_T3′) and d(5′TG3′-3′G_2_T5′), and the parent d(TG_3_T) were subject of attempts to fold into G-quadruplex structures upon titration of ^15^NH_4_Cl into aqueous solutions of carefully dialyzed oligonucleotides. At 25°C single strands have been observed at 10 mM concentration of ^15^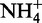 ions almost exclusively, except for oligonucleotide d(3′TG5′-5′G_2_T3′). Decrease of temperature to 0°C has resulted immediately in well-resolved imino (Figure 1a and b), aromatic and other proton resonances in ^1^H NMR spectra of d(TG_3_T) and d(3′TG5′-5′G_2_T3′). In the case of d(3′T5′-5′G_3_T3′) and d(5′TG3′-3′G_2_T5′), the majority of the oligonucleotides did not fold into quadruplex structures even at 0°C (Supplementary Figure S1). Increase of ^15^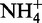 ion concentration to 80 mM has led to almost complete formation of G-quadruplex structures at 0°C for all four studied oligonucleotides (Figure 1c–f). Despite higher concentration of ^15^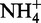 ions, increase of temperature to 25°C resulted in disintegration of the G-quadruplex structures by 90% in the case of d(3′T5′-5′G_3_T3′), d(5′TG3′-3′G_2_T5′) and the parent d(TG_3_T) (data not shown) and thus all further measurements were made at 0°C.

At 10 mM concentration of ^15^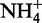 ions, two sets of observed ^1^H NMR signals in the case of [d(TG_3_T)]_4_ indicate the formation of two species with different populations. The ratio between the major and minor forms of [d(TG_3_T)]_4_ quadruplexes was 60:40 (Figure 1a). It is interesting to note that sharp imino proton resonances of thymine residues at both 5′- and 3′-ends have been observed in ^1^H NMR spectra, which are indicative of their protection against exchange with bulk water. Imino protons of thymine residues at the 3′-end exhibit almost isochronous chemical shifts in major and minor forms of [d(TG_3_T)]_4_. In contrast, imino protons of thymine residues at the 5′-end exhibit different chemical shifts thus indicating that the 5′-ends of the two forms exhibit distinctive shielding caused by local structural differences. At 80 mM salt concentration, resolved imino protons have been observed only for G2* and G3* of the minor form of [d(TG_3_T)]_4_ (Figure 1c). On the other hand, imino signals of G2 and G3 residues in the major form are probably buried in the crowded region between *δ* 10.90 and 11.20 ppm at 80 mM ^15^NH_4_Cl. In the case of [d(3′TG5′-5′G_2_T3′)]_4_ quadruplex, the comparison of spectral characteristics at 10 and 80 mM concentrations of ^15^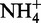 ions indicates negligible influence of salt concentration on G-quadruplex formation (Figure 1b and d). The ratio of integrals of imino resonances of G3 and G4 in the two forms (overlapped between *δ* 11.40 and 11.60 ppm) with respect to G2 of one of the forms is ca. 4:1. The observed ratio between imino protons suggests formation of two equally populated symmetric G-quadruplex structures, where G2* residues in one of the forms are less protected from imino proton exchange, signifying formation of a more flexible G-quartet. Imino signals of T5 residues have been observed for both forms of [d(3′TG5′-5′G_2_T3′)]_4_ as a single peak, whereas unambiguous assignment of imino signals of T1 has been made only for form consisting of G2 quartet. The signal with chemical shift of *δ* 10.96 ppm could be tentatively assigned to imino protons of T1*. The comparison of resonance line widths of imino signals of T1 and T1* and respective intensities suggest decreased rigidity of the part of G-quadruplex structure consisting of T1* and G2* residues. Unfortunately, additional minor signals could not be assigned to the specific structure(s).

Although in the presence of 10 mM concentration of ^15^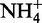 ions, d(3′T5′-5′G_3_T3′) and d(5′TG3′-3′G_2_T5′) form only barely observable amounts of G-quadruplex structures (Supplementary Figure S1c and S1d), increase of ^15^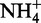 ion concentration to 80 mM results in several sets of imino signals of various intensities, thus indicating formation of multiple G-quadruplex species with different populations (Figure 1e and f). In both cases, three distinctive G-quadruplex species have been identified, while additional minor signals are present that could not be assigned. It is interesting to note that our imino proton assignments of major forms are in agreement with assignments of the same G-quadruplex structures in the presence of K^+^ ions (15). Imino proton assignments of less populated forms were additionally confirmed by analyzing correlations between imino protons and bound ^15^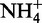 ions (*vide infra*).

The aromatic-anomeric region of 2D NOESY spectrum of d(TG_3_T) in the presence of 10 mM concentration of ^15^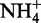 ions clearly ascertained double set of signals for almost all resonances (Supplementary Figure S2a). A continuous 5′-3′ sequential NOE connectivity pattern has been found for the major form that is typical of G-quadruplex structure in which all residues adopt *anti* conformations. Interestingly, even though almost all cross-peaks in aromatic-anomeric region are in duplicates, the major and minor forms of [d(TG_3_T)]_4_ show different sequential NOE connectivities and therefore distinctive structural features. Remarkably, the inversion of polarity sites in d(3′T5′-5′G_3_T3′) leads to formation of G-quadruplex form with almost identical sequential connectivities in the aromatic-anomeric regions of 2D NOESY spectrum as have been observed for the minor form of the parent sequence (Supplementary Figures S2a and S4a). Taking into account that a *syn* conformation has been ascertained for residue G2 in the major form of [d(3′T5′-5′G_3_T3′)]_4_ (*vide infra*), the minor form of [d(TG_3_T)]_4_ consists of two G-quartets with all residues in an *anti* conformation and one G-quartet (formed by G2* residues) adopting a *syn* conformation. At 80 mM concentration of ^15^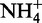 ions, complete sequential H8-H1′ NOE walk has been observed only for the minor form of [d(TG_3_T)]_4_ (Supplementary Figure S2b). For [d(3′TG5′-5′G_2_T3′)]_4_ quadruplex, identical sequential H8-H1′ NOE connectivities have been observed at 10 and 80 mM cation concentrations (Supplementary Figure S3a and S3b). The perusal of aromatic-anomeric and aromatic-H2′/H2′′ regions of 2D NOESY spectra of [d(3′TG5′-5′G_2_T3′)]_4_ quadruplex clearly ascertains that residue G3 adopts *syn* conformation, while all other G residues are in *anti* conformation (Supplementary Figure S3). The presence of the 5′-5′ inversion of polarity site prevented us to perform a complete sequential walk in [d(3′TG5′-5′G_2_T3′)]_4_. However, typical sequential NOE connectivities for *syn-anti* step can be nicely seen between G3 and G4 residues. Analysis of NOESY spectra of [d(3′T5′-5′G_3_T3′)]_4_ in the presence of 80 mM concentration of ^15^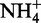 ions clearly ascertained that G2 residues adopted a *syn* conformation, while all other G nucleotides were in *anti* conformation (Supplementary Figure S4). The inversion of polarity site between T1 and G2 residues enabled observation of continuous sequential NOE connectivities only from G2 to T5 residues in [d(3′T5′-5′G_3_T3′)]_4_. Because of the inversion of polarity sites, [d(5′TG3′-3′G_2_T5′)]_4_ at 80 mM ^15^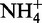 ion concentration consists of two 5′-3′ segments in antiparallel orientation. For each segment, separate NOE sequential walks have been identified for all forms consisting of residues with only *anti* conformation around glycosidic bond (Supplementary Figure S5).

### Localization of ^15^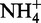 ions within the canonic [d(TG_3_T)]_4_ quadruplex

**Figure 2.**
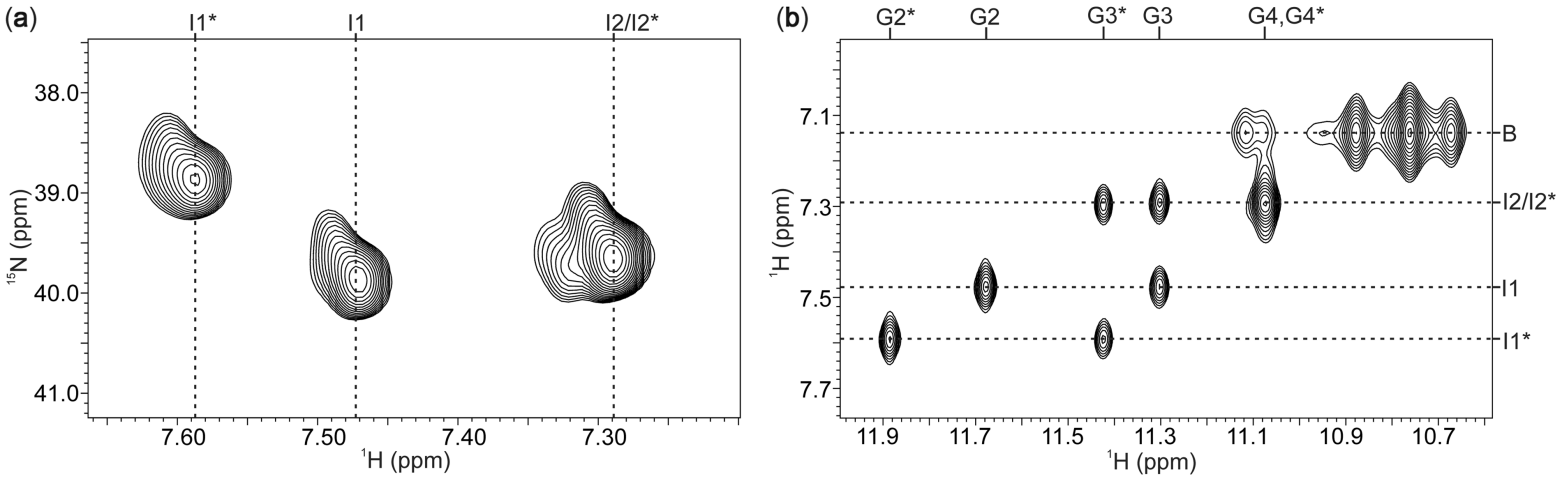
^15^N-^1^H HSQC (**a**) and selected NOESY spectra (*τ*_m_ = 80 ms) (b) of G-quadruplexes adopted by d(TG_3_T) in the presence of 10 mM ^15^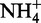 ions at 0°C. Cross-peaks corresponding to ^15^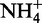-ion-binding sites in the minor form are labeled with stars. The label ‘/’ indicates cross-peaks of the two forms with isochronous chemical shifts along ^1^H and ^15^N dimensions. In panel (**b**), labels along the imino region correspond to the assigned guanine residues in the two forms. The label B indicates ^15^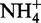 ions in bulk solution.

**Figure 3.**
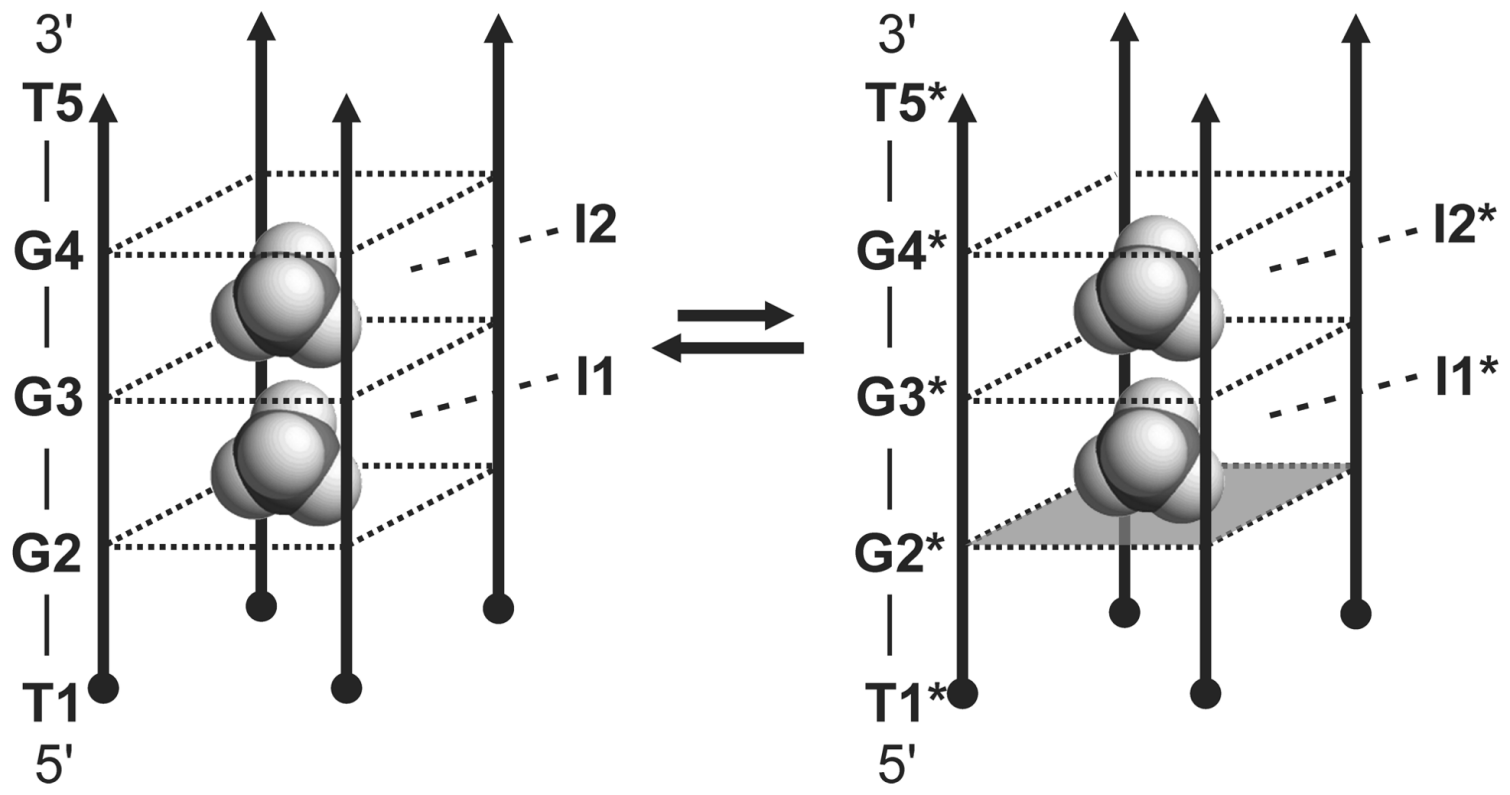
Equilibrium between the major (left) and the minor (right) G-quadruplex forms adopted by d(TG_3_T) at 10 mM concentration of ^15^NH_4_Cl. Individual residues are noted along one of the strands of each form. The labels with stars indicate residues and ^15^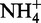-ion-binding sites of the minor form. Black circles and arrowheads indicate the 5′- and 3′-ends of each strand, respectively. G-quartet with all-*syn* residues is depicted as a gray square.

The 2D NOESY and ^15^N-^1^H HSQC experiments were used to establish ^15^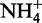-ion-binding sites within G-quadruplexes formed by d(TG_3_T) in the presence of 10 mM concentration of salt. Analysis of the ^15^N-^1^H HSQC spectra has revealed three resolved cross-peaks that correspond to ^15^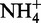 ions in different chemical environments (Figure 2a). The ratio of volumes of HSQC cross-peaks corresponding to sites I1*, I1 and I2/I2* was 1.0, 0.8 and 2.3 indicating different occupancies at individual binding sites in the two G-quadruplex forms, respectively. Localization of ions was possible through analysis of NOE connectivities between ^15^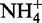 ion protons and neighboring imino protons of guanine bases involved in G-quartets in both forms of [d(TG_3_T)]_4_ (Figure 2b). ^15^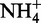 ions resonating at *δ* 7.48 ppm are localized between G2 and G3 quartets (binding site I1) in the major form of [d(TG_3_T)]_4_. ^15^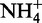 ions with chemical shift of *δ* 7.29 ppm are localized between G3 and G4 quartets in the major and in the minor forms and are therefore assigned to binding sites I2 and I2* (Figure 3). ^15^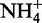 ions resonating at *δ* 7.29 ppm (I2*) and *δ* 7.59 ppm (I1*) both show cross-peaks to imino protons of G3* at *δ* 11.42 ppm in the minor form of [d(TG_3_T)]_4_ (Figure 2b). In addition, ^15^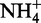 ions at binding site I2* exhibit a cross-peak with imino protons of G4*, whereas ^15^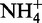 ions localized at binding site I1* display a cross-peak to imino proton of G2* (Figure 2b). It is interesting to note that in the minor form of [d(TG_3_T)]_4_, binding site I1* is between G2*-quartet consisting of all residues in a *syn* and G3*-quartet with all residues in an *anti* conformation around glycosidic bonds. At 80 mM concentration of ammonium salt, the cross-peaks indicating ^15^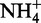-ion-binding sites within the minor form of [d(TG_3_T)]_4_ were observed at almost the same chemical shifts as described earlier. On the other hand, signals corresponding to ^15^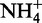 ions within the major form exhibited upfield chemical shift changes. Because of poor dispersion and broad nature of signals, further evaluation of ^15^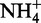-ion-binding sites within the major form of [d(TG_3_T)]_4_ was hampered at higher cation concentration.

### Localization of ^15^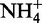 ions within G-quadruplexes with inversion of polarity sites

**Figure 4.**
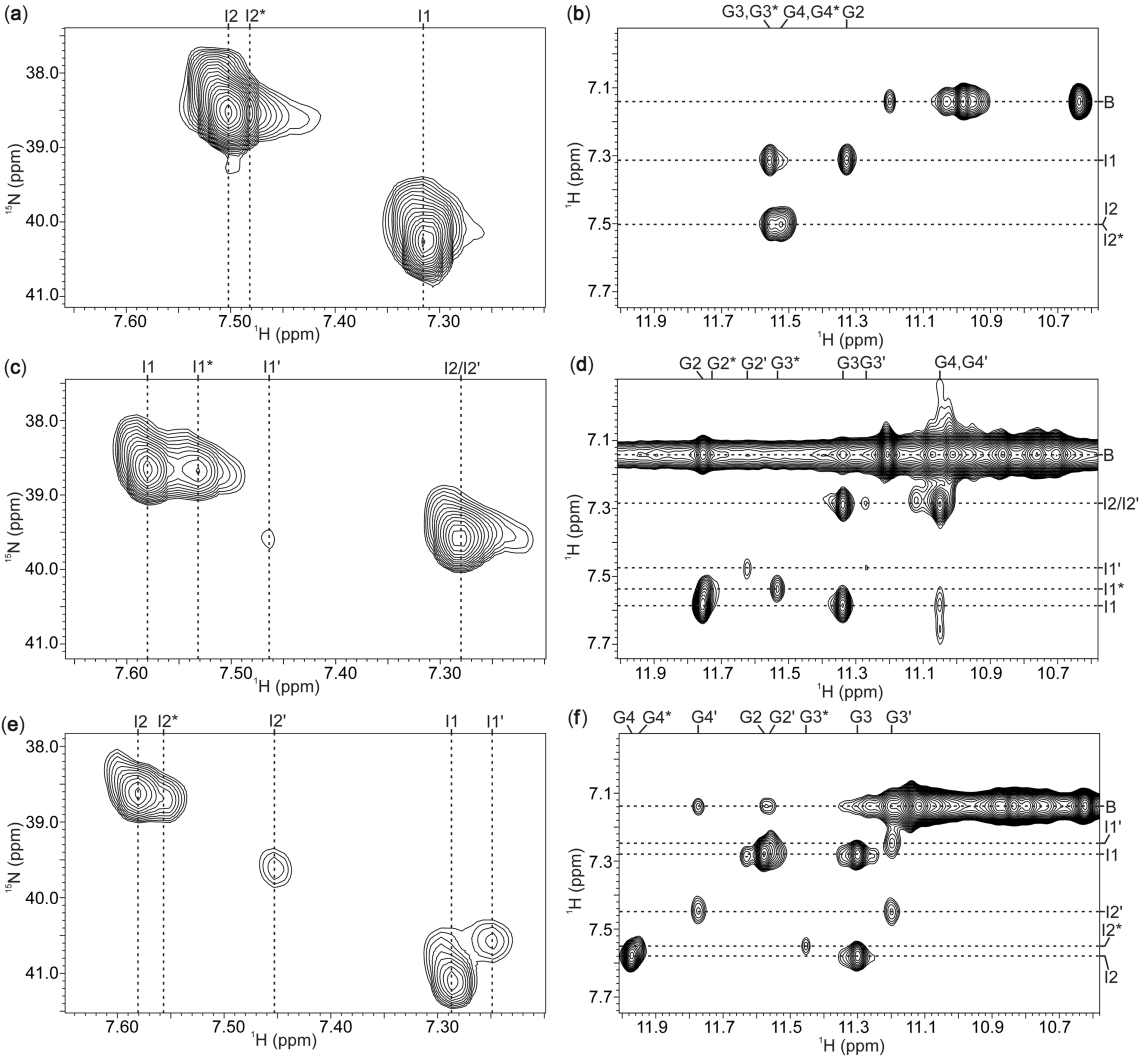
^15^N-^1^H HSQC and NOESY spectra (*τ*_m_ = 150 ms) of G-quadruplex adopted by d(3′TG5′-5′G_2_T3′) (**a**, **b**) d(3′T5′-5′G_3_T3′) (**c**, **d**) and d(5′TG3′-3′G_2_T5′) (**e**, **f**) in the presence of 80 mM ^15^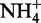 ions at 0°C. ^15^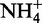-ion-binding sites and residues of the minor forms are marked with superscripts (* and ′). The label B indicates ^15^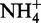 ions in bulk solution.

**Figure 5.**
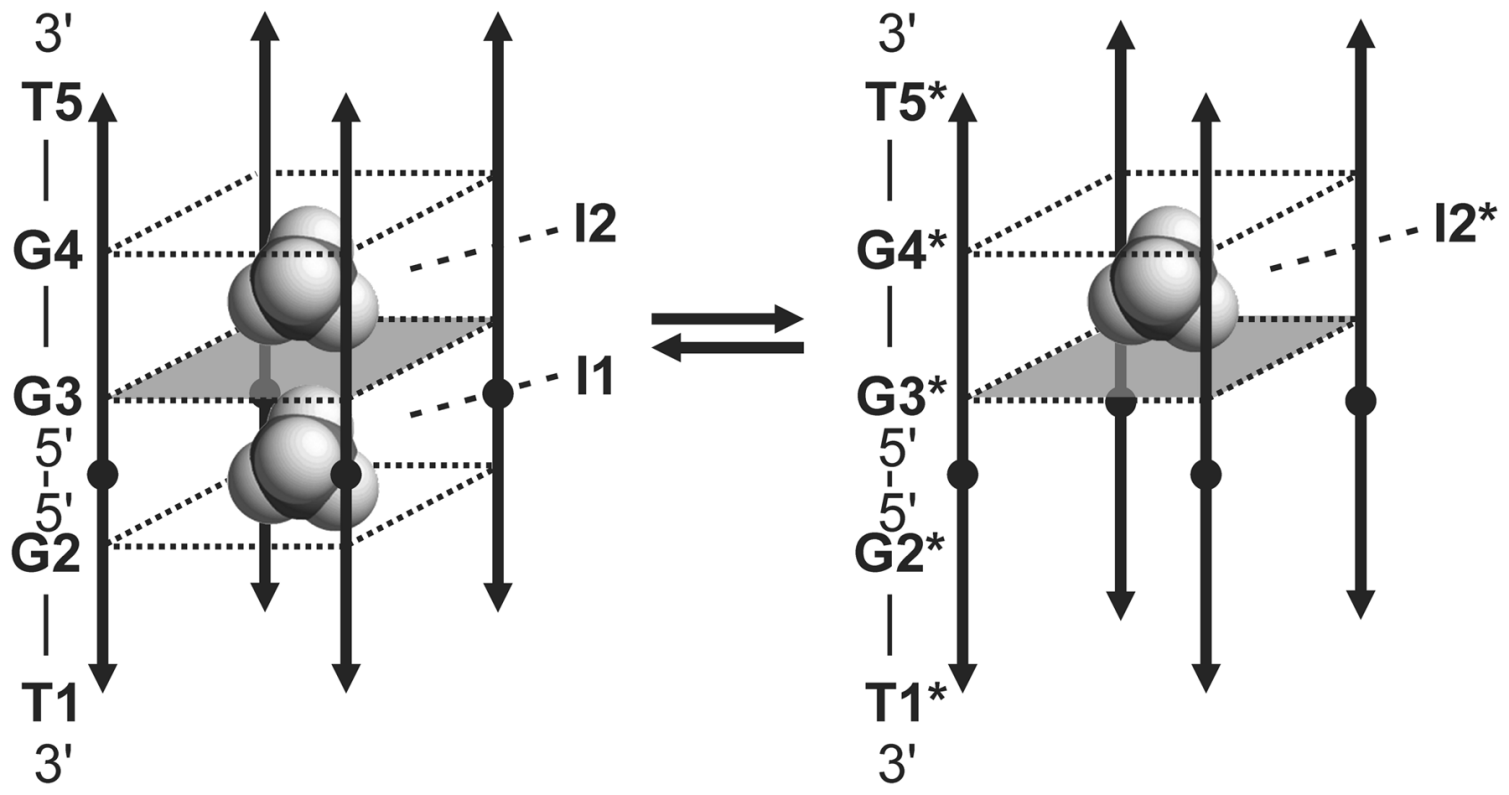
Equilibrium between the two G-quadruplex forms adopted by d(3′TG5′-5′G_2_T3′). Individual residues are indicated along one of the strands. ^15^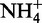-ion-binding sites in the interior of both forms are labeled. The labels with stars correspond to the form with only a single-cation-binding site. Black circles and arrowheads indicate the 5′- and 3′-ends of each strand, respectively. G-quartet with all-*syn* residues is depicted as a gray square. More flexible G-quartet consisting of G2* residues is not marked as a square.

**Figure 6.**
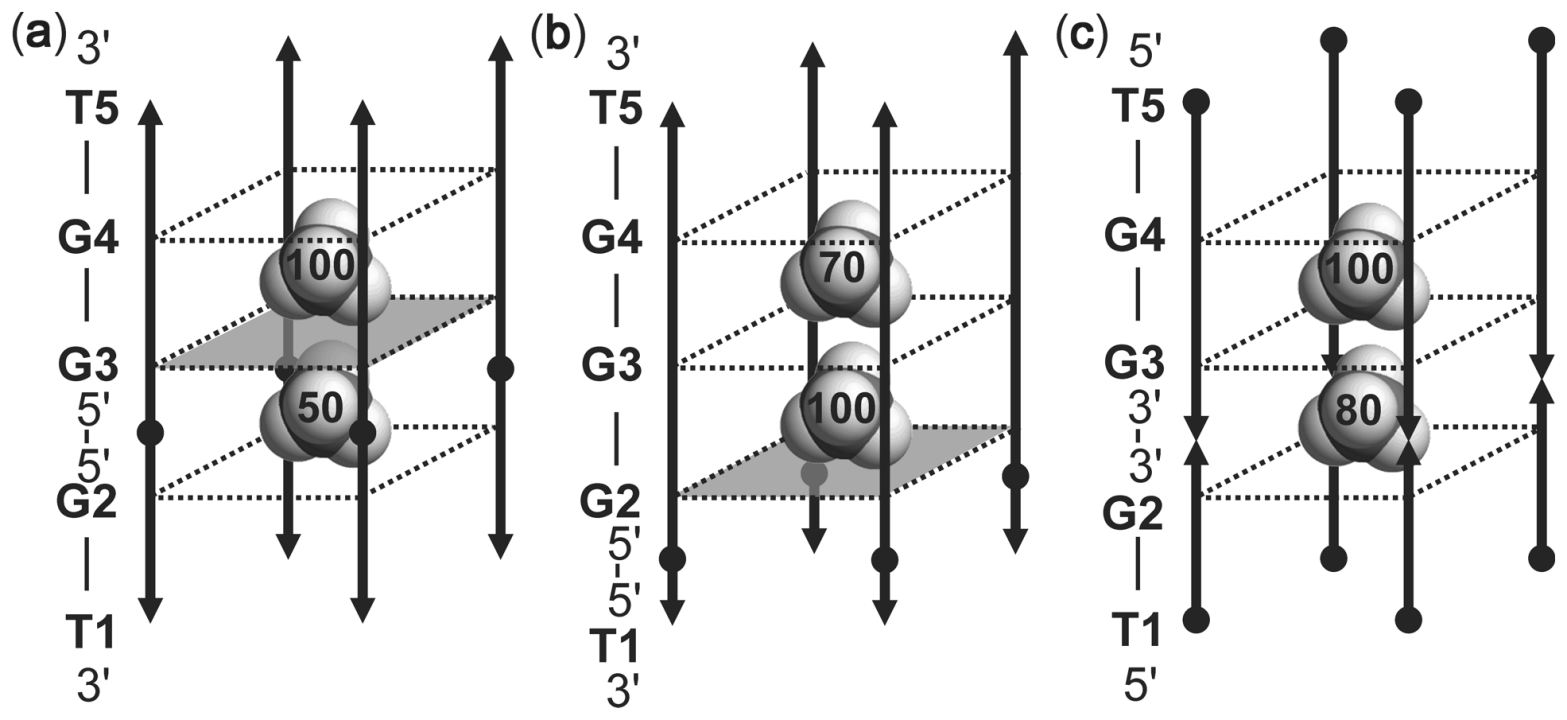
Relative occupancies of individual binding sites (expressed as % of the most populated binding site) of [d(3′TG5′-5′G_2_T3′)]_4_ (**a**), [d(3′T5′-5′G_3_T3′)]_4_ (**b**) and [d(5′TG3′-3′G_2_T5′)]_4_ (**c**) quadruplexes. Shaded squares in (a) and (b) represent G-quartets with residues in a *syn* conformation.

^15^N-^1^H HSQC spectra of [d(3′TG5′-5′G_2_T3′)]_4_ in the presence of 10 and 80 mM of ^15^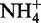 ions exhibited two resolved cross-peaks corresponding to ^15^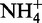 ions at different binding sites inside G-quadruplex (Figure 4a and Supplementary Figure S6). NOESY spectrum of [d(3′TG5′-5′G_2_T3′)]_4_ exhibits cross-peaks between ^15^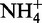 ions resonating at ^1^H chemical shift of *δ* 7.32 ppm (I1) with G2 and G3 imino protons (Figure 4b). ^15^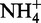 ions with the ^1^H chemical shift of *δ* 7.50 ppm show NOE contacts to imino protons of G3 and G4 residues (binding site I2 in Figures 4b and 5). It is interesting to note that cross-peak corresponding to ^15^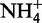 ions at binding site I2 exhibits a smaller, partially overlapped cross-peak on its upfield side along ^1^H axis (Figure 4a and b). This observation is attributed to the presence of two different G-quadruplex forms in solution that are in intermediate exchange on NMR time scale. The respective cross-peak volumes corresponding to ^15^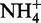 ions at three distinct binding sites in two forms of [d(3′TG5′-5′G_2_T3′)]_4_ are identical. One of the forms thus exhibits two bound cations between three G-quartet planes, whereas the other form exhibits only one cation-binding site between G-quartets composed of G3* and G4* residues (Figure 5). Quantitative assessment shows that approximately 50% of the G-quadruplex structures bind only one cation. Therefore, NMR data suggest that the cation-binding site, where the inversion of polarity does not occur, is the preferred binding site for cations in [d(3′TG5′-5′G_2_T3′)]_4_ quadruplex because it is occupied by ^15^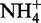 ions inside both forms (Figure 6a).

According to the observation that d(3′T5′-5′G_3_T3′) in the presence of 80 mM concentration of ^15^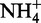 ions folded into more than one G-quadruplex form (Figure 1e), its ^15^N-^1^H HSQC spectrum exhibited three larger and one smaller cross-peak (Figure 4c). The 2D NOESY spectrum made it possible to localize cations inside [d(3′T5′-5′G_3_T3′)]_4_ G-quadruplex. ^15^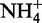 ions with ^1^H chemical shifts of *δ* 7.58 (I1) and *δ* 7.28 ppm (I2) both exhibit NOE contacts to imino protons of G3 residues with *δ* 11.34 ppm (Figure 4d). In addition, the former also show NOE contacts to imino protons of G2 residues, whereas the latter exhibit NOE contacts to imino protons of G4 residues in the major (ca. 60%) form of [d(3′T5′-5′G_3_T3′)]_4_. Furthermore, NMR data point to the presence of another form of [d(3′T5′-5′G_3_T3′)]_4_ in smaller quantity of approximately 10%, that also binds two cations with ^1^H chemical shifts of *δ* 7.46 ppm (I1′, binding site between G-quartets composed of G2′ and G3′ residues) and of *δ* 7.28 ppm (I2′, binding site between G-quartets composed of G3′ and G4′ residues). ^15^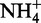 ions resonating at ^1^H chemical shift of *δ* 7.53 ppm (I1*) exhibit NOE cross-peaks with G2* and G3* imino protons (Figure 4d). It is interesting to note that this form with population of approximately 30% exhibits only a single-cation-binding site between G-quartets composed of G2* and G3* residues. Cation-binding site placed between the middle G-quartet and G-quartet composed of guanine residues in *syn* conformation is thus occupied by ^15^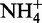 ions in all identified forms of [d(3′T5′-5′G_3_T3′)]_4_ (Figure 6b). Based on the number of imino signals in ^1^H NMR spectrum, formation of more than one G-quadruplex form was observed for d(5′TG3′-3′G_2_T5′) at 80 mM of ^15^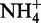 ions (Figure 1f). Perusal of 2D NOESY and ^15^N-^1^H HSQC spectra revealed formation of three different G-quadruplex forms of [d(5′TG3′-3′G_2_T5′)]_4_ (Figure 4e and f). Two forms bind two cations each, while one of the form exhibit a single-cation-binding site. The major form with population of ca. 60% binds two ^15^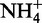 ions with the ^1^H chemical shifts of *δ* 7.58 (I2) and *δ* 7.29 ppm (I1) that both show NOE contacts to imino protons of G3 residues (Figure 4f). ^15^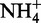 ions at binding sites I1 and I2 in addition exhibit dipole–dipole correlations to imino protons of G2 and G4 residues, respectively. ^15^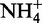 ions with the ^1^H chemical shifts of *δ* 7.45 (I2′) and *δ* 7.25 ppm (I1′) are bound within the other form of [d(5′TG3′-3′G_2_T5′)]_4_ with two cations (ca. 20% relative population). ^15^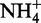 ions at binding site I2′ show correlations to imino protons of G3′ and G4′ residues in NOESY spectrum, whereas ^15^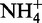 ions at binding site I1′ show NOE cross-peaks to imino protons of G2′ and G3′ residues (Figure 4f). ^15^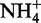 ions localized inside G-quadruplex form with only one cation-binding site (ca. 20% relative population) resonate at ^1^H chemical shifts of *δ* 7.55 (I2*) ppm. They exhibit NOE correlations to neighboring imino protons of G3* and G4* residues (Figure 4f). Therefore, the cation-binding site where inversion of polarity sites does not occur in [d(5′TG3′-3′G_2_T5′)]_4_ is occupied by ^15^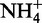 ions inside all three forms (Figure 6c).

### Cation movements from/into the canonical quadruplex [d(TG_3_T)]_4_: influence of an all-syn G-quartet

**Figure 7.**
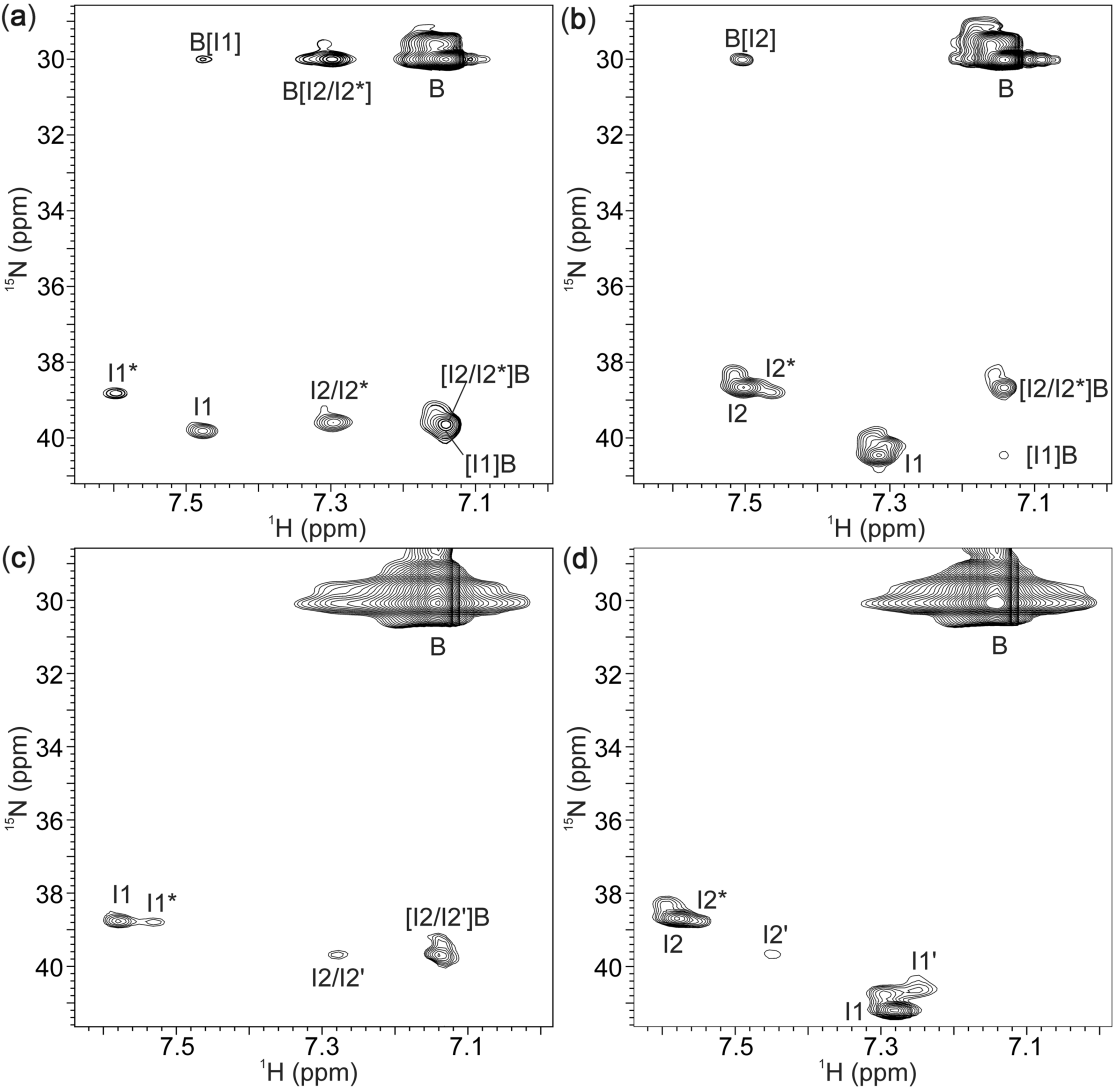
The 2D ^15^N-^1^H NzExHSQC spectra of [d(TG_3_T)]_4_ (**a**), [d(3′TG5′-5′G_2_T3′)]_4_ (**b**), [d(3′T5′-5′G_3_T3′)]_4_ (**c**) and [d(5′TG3′-3′G_2_T5′)]_4_ (**d**) at 0°C in 10% ^2^H_2_O. Concentration of ^15^NH_4_Cl was 10 mM in (a, b) and 80 mM in (c, d). Mixing times were 1.3 s (a), 0.5 s (b) and 0.3 s (c, d). The autocorrelation and cross-peaks are labeled. The cross-peak labels consist of two parts, where the part in square brackets indicates binding sites within G-quadruplexes. The first part of a label indicates the initial position, whereas the second part corresponds to a final site of cation movement. The label B indicates ^15^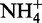 ions in bulk solution.

The occurrence of cross-peaks in addition to autocorrelation peaks in the 2D ^15^N-^1^H NzExHSQC spectra clearly demonstrates the movements of ^15^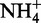 ions between identified binding sites and bulk solution (Figure 7a). ^15^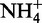 ion movement from interior (binding sites I2 and I2* in the major and in the minor forms, respectively) of G-quadruplex structure into bulk solution is characterized by a single cross-peak [I2/I2*]B. Cross-peak [I1]B indicates movement of ^15^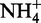 ions from binding site I1 into bulk solution. Unfortunately, cross-peaks [I2/I2*]B and [I1]B are severely overlapped, and directionality of the preferred ammonium ion movement along the central cation cavity cannot be evaluated. The reverse movement from bulk solution into binding sites I2 and I2* is characterized by an intense cross-peak B[I2/I2*]. Weak cross-peak B[I1] is a result of ion movement from bulk solution into binding site I1. It is interesting to note that no cross-peak corresponding to cation movements between binding site I1* and bulk solution through G-quartet with *syn* residues has been observed. In both forms of [d(TG_3_T)]_4_, ammonium ions are not exchanging between the two inner binding sites. Rate constants for movements can be directly determined by the evaluation of cross-peak intensities as a function of mixing time of ^15^N-^1^H NzExHSQC experiment (23). Quantitative analysis of overlapped [I2/I2*]B and [I1]B cross-peaks afforded rate constant k_[I2/I2*]B+[I1]B_ of 1.1 ± 0.1 s^−^^1^ at 0°C. The ^15^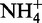 ion movement from bulk solution into G-quadruplex at the 3′-end of major and minor forms is characterized by rate constant k_B[I2/I2*]_ of 1.0 ± 0.1 s^−^^1^ at 0°C. Movement into binding site I1 at the 5′-end of the major form is characterized by rate constant k_B[I1]_ of 0.2 ± 0.1 s^−^^1^ at 0°C. The values indicate that exchange of ^15^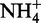 ions between G-quadruplex and bulk solution is ca. 5 times faster at the 3′-end in comparison to the 5′-end.

### Cation movements between bulk solution and G-quadruplex with inversion of polarity sites

The movement of ^15^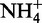 ions from binding sites I2 and I2* of [d(3′TG5′-5′G_2_T3′)]_4_ into bulk solution is demonstrated by single cross-peak [I2/I2*]B. The movement from bulk to binding site I2 is defined by the cross-peak B[I2] (Figure 7b). The weak cross-peak [I1]B indicates cation movement from binding site I1 into bulk solution. Quantification of [I2/I2*]B cross-peak volume as a function of mixing time afforded an apparent rate constant of 1.4 ± 0.1 s^−^^1^ at 10 mM and 1.6 ± 0.2 s^−^^1^ at 80 mM salt concentration. Thus, taking into account the experimental error, ^15^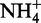 ion concentration has little if any influence on rates of movements from binding sites I2 and I2* into bulk solution. Movement from bulk solution into binding site I2 in [d(3′TG5′-5′G_2_T3′)]_4_ is characterized by rate constant of 0.7 ± 0.1 s^−^^1^ at 10 and 80 mM concentration of ^15^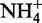 ions. At lower concentration of salt, movement from binding site I1, where the inversion of polarity site takes place, is characterized by rate constant of 0.4 ± 0.1 s^−^^1^. On the other hand, at 80 mM salt concentration, ^15^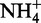 ion movement from binding site I1 to bulk solution is slower considerably because no cross-peak has been observed till the mixing time of 3 s (Supplementary Figure S7).

Perusal of ^15^N-^1^H NzExHSQC spectra of [d(3′T5′-5′G_3_T3′)]_4_ in the presence of 80 mM concentration of ^15^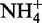 ions reveals cross-peak corresponding to the movement of ^15^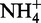 ions from binding sites I2 and I2′ into bulk solution. Quantitative analysis of [I2/I2′]B cross-peak afforded the rate constant k_[I2/I2__′__]B_ of 10 ± 1 s^−^^1^ at 0°C (Figure 7c). Reverse movement from bulk solution into the binding sites within different G-quadruplex forms could not be observed by a resolved cross-peak. ^15^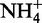 ion movement from the binding sites I1, I1′ and I1* into bulk solution could not be observed for [d(3′T5′-5′G_3_T3′)]_4_ quadruplex. No cross-peaks indicating cation movements have been observed in NzExHSQC spectra of [d(5′TG3′-3′G_2_T5′)]_4_ (Figure 7d). In addition, it is interesting to note that no cross-peaks, which would indicate movements of cations between the two inner binding sites within [d(3′TG5′-5′G_2_T3′)]_4_, [d(3′T5′-5′G_3_T3′)]_4_ and [d(5′TG3′-3′G_2_T5′)]_4_ were observed.

## DISCUSSION

In the presence of 10 mM concentration of ^15^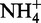 ions at 0°C, d(TG_3_T) folds into two G-quadruplex structures. Its modified analogs containing an inversion of polarity sites exhibit greatly reduced G-quadruplex formation at this cation concentration. However, d(3′TG5′-5′G_2_T3′) with a 5′-5′ inversion in the G-run forms stable quadruplex structures under the same conditions. The increase of ^15^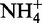 ion concentration to 80 mM resulted in G-quadruplex formation also for d(3′T5′-5′G_3_T3′) and d(5′TG3′-3′G_2_T5′) at 0°C. At 25°C, only G-quadruplex formed by d(3′TG5′-5′G_2_T3′) was stable. In all the other ODNs, reduction of amount of G-quadruplex structure by 90% has been observed upon increase of temperature from 0 to 25°C. A comparison of behaviors of d(3′TG5′-5′G_2_T3′) and d(5′TG3′-3′G_2_T5′) in the presence of ^15^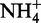 ions demonstrates a better aptitude in stabilization of G-quadruplexes comprising a 5′-5′ inversion of polarity site in a G-run than a 3′-3′, because only in the first case, a stable G-quadruplex structure could be observed at lower salt concentration and higher temperature. In full agreement, the apparent melting temperature of related constructs was 38°C higher in d(3′TG_2_5′-5′G_2_T3′) with respect to d(5′TG_2_3′-3′G_2_T5′) (12). The introduction of a 5′-5′ inversion of polarity site into a G-run clearly points toward remarkable enhancement of the thermal stability in comparison to the natural counterpart (15). In contrast, a 5′-5′ inversion of polarity site between T and G residues in d(3′T5′-5′G_3_T3′) reduces the level of formation of G-quadruplexes with respect to d(3′TG5′-5′G_2_T3′) at the same experimental conditions.

The parent d(TG_3_T) forms two distinct G-quadruplex structures with the ratio of 60:40 that are in slow exchange on the NMR time scale in the presence of 10 mM of ^15^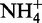 ions. The core of both structures consists of three G-quartets. However, the major form consists of a canonical tetramolecular parallel G-quadruplex in which all guanine residues adopt an *anti* conformation, whereas in the minor form, the G-quartet at the 5′-end exhibits all guanine residues in a *syn* conformation. Similarly, the minor form of [d(TG_4_T)]_4_ also contains an all-*syn* G-quartet consisting of G2 residues, which was recently misassigned due to its low population (27). The resonance assignment of the minor species of [d(TG_3_T)]_4_ was achieved by comparison of a NOESY spectrum of [d(TG_3_T)]_4_ with that of [d(3′T5′-5′G_3_T3′)]_4_, which adopts almost exclusively a structure where G2 residues form an all-*syn* G-quartet. The first direct evidence of the occurrence of an all-*syn* G-quartet in a parallel quadruplex was in [d(TG^Me^GGT)]_4_ where G^Me^ is an 8-methyl-2′-deoxyguanosine (31). In this case, an all-*syn* G-quartet was probably favored by the substitution at C8 of guanine. Several kinetic studies concerning tetramolecular parallel quadruplexes reported that the presence of an 8-substituted guanine at the 5′-end of the G-run is able to accelerate quadruplex formation (32,33). Based on these findings, it has been hypothesized that a *syn* dG at the 5′-end of a strand is involved in the nucleation process that initiates quadruplex association (32). To the best of our knowledge, we report herein the first experimental evidence of guanine residues in a *syn* conformation within tetramolecular parallel quadruplex that is not induced by any structural substitutions. This form could represent the early state in the assembly process of [d(TG_3_T)]_4_. Interestingly, one of the sequential steps in its structure is a *syn-anti* that recent MD simulations have suggested to be energetically preferred over an *anti-anti* step in G-quadruplexes (34).

The inversion of polarity site in d(3′TG5′-5′G_2_T3′) results in two equally populated tetramolecular parallel-like G-quadruplex forms in the presence of ^15^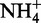 ions. One of the forms is a G-quadruplex consisting of the three G-quartets, where G3 residues exhibit a *syn* conformation, whereas G2 and G4 are in an *anti* conformation. In the other form, G2* residues are involved in a more flexible G-quartet. It is interesting to note that, contrarily to a parallel-like G-quadruplex structure adopted by d(3′TG5′-5′G_2_T3′), the related oligonucleotide sequence d(3′TG5′-5′G_3_T3′), with an additional guanine residue, possesses a 2-fold symmetry, where a tetrameric antiparallel quadruplex is embedded between two parallel strands (14). On the other hand, d(3′TG_2_5′-5′G_2_T3′), where a 5′-5′ inversion of polarity site occurs in the middle of a G-run, maintains a parallel-like quadruplex structure (12). In the case of d(3′T5′-5′G_3_T3′) and d(5′TG3′-3′G_2_T5′) in the presence of 80 mM concentration of ^15^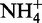 ions, three different G-quadruplex forms have been identified. All forms of [d(5′TG3′-3′G_2_T5′)]_4_ consist of three G-quartets with all guanine residues in an *anti* conformation. In contrast, all forms of [d(3′T5′-5′G_3_T3′)]_4_ contain an all-*syn* G2-quartet in addition to two G-quartets in an *anti* conformation.

Each of the [d(TG_3_T)]_4_ quadruplex forms binds two ^15^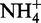 ions. Both binding sites in the major form are between two all-*anti* G-quartets, whereas one of the two binding sites in the minor form is between an all-*syn* and an all-*anti* G-quartets. [d(3′TG5′-5′G_2_T3′)]_4_ with three G-quartets also binds two ^15^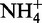 ions each between an all-*syn* and an all-*anti* G-quartets. The form of [d(3′TG5′-5′G_2_T3′)]_4_ with the lower ^15^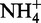 ion occupancy exhibits only a single cation-binding site between G-quartets formed by G3* and G4* residues. d(3′T5′-5′G_3_T3′) and d(5′TG3′-3′G_2_T5′) form two structures binding two cations and one structure with only a single-cation-binding site. In the structures with a single cation, ^15^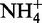 ions are located between the middle G-quartet and the G-quartet formed by G2* residues in [d(3′T5′-5′G_3_T3′)]_4_ and between the middle G-quartet and the G-quartet formed by G4* residues in [d(5′TG3′-3′G_2_T5′)]_4_. Hence, the inversion of polarity sites has a great influence on occupancy of cation-binding sites. If inversion of polarity sites is within a G-run like in [d(3′TG5′-5′G_2_T3′)]_4_ and [d(5′TG3′-3′G_2_T5′)]_4_, the binding sites involving natural 5′-3′ backbone exhibit higher occupancy in both structures in comparison to the modified site involving 3′-3′ or 5′-5′ sugar-phosphate backbone. On the other hand, if inversion of polarity sites occurs outside a G-run like in [d(3′T5′-5′G_3_T3′)]_4_, all-*syn* G-quartet shows a great influence on the occupancy of binding sites by cations. Thus, binding site with higher population of cations is between one all-*syn* and one all-*anti* G-quartet.

The comparison of rate constants for the movement of ^15^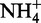 ions from individual binding site in the major form of [d(TG_3_T)]_4_ to bulk solution and back reveals faster movements of cations from the binding site closer to the T5 residues (e.g. I2 binding site). The comparative analysis suggests that cation movement from tetramolecular G-quadruplexes into bulk solution is faster from the canonical 5′-3′ sites closer to the 3′-end. In complete agreement, in [d(5′TG3′-3′G_2_T5′)]_4_ quadruplex where both end residues, T1 and T5, are at the 5′-ends, no cation movement have been observed from G-quadruplex into bulk solution. Beside strand directionality, cation movement is also influenced by the features of an all-*syn* G-quartet. In the case of the minor form of [d(TG_3_T)]_4_, the formation of an all-*syn* G-quartet consisting of G2 residues exhibits great influence on cation movement from binding site closer to T1 residues into bulk solution. In fact, no cation movement has been observed from that binding site to bulk solution. In addition, no cation movement has been observed also from binding site closer to T1 within [d(3′T5′-5′G_3_T3′)]_4_ quadruplex, that, similarly to the minor form of [d(TG_3_T)]_4_ is placed between an all-*syn* G-quartet and an all-*anti* G-quartet. However, in the case of [d(3′T5′-5′G_3_T3′)]_4_, quadruplex T1 residues are closer to the 3′-end due to the inversion of polarity site. It is interesting to note that cation movement from the inter-quartet binding sites consisting of 5′-3′ sugar-phosphate backbone (e.g. I2 binding site) of [d(3′TG5′-5′G_2_T3′)]_4_ and [d(3′T5′-5′G_3_T3′)]_4_ to bulk solution is an order of magnitude slower in the former, where the binding site I2 is placed between one all-*anti* and one all-*syn* G-quartet rather than two all-*anti* G-quartets. The current data suggest that an all-*syn* G-quartet represents a major hindrance for cation movement instead of a T-quartet as was proposed recently (27). Even though the [d(TG_3_T)]_4_, [d(3′TG5′-5′G_2_T3′)]_4_, [d(3′T5′-5′G_3_T3′)]_4_ and [d(5′TG3′-3′G_2_T5′)]_4_ quadruplex structures involve two cation-binding sites for ^15^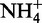 ions, no cation movements between the inner binding sites have been observed. Similarly, for a dimeric quadruplex structure [d(G_3_T_4_G_4_)]_2_, exhibiting two cation-binding sites between three G-quartet planes, ^15^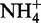 ion movements have been observed only between each of the binding sites and the bulk solution and not between the binding sites (23). It seems a general feature that cation movement is decelerated, if present at all, among the inner binding sites within G-quadruplexes consisting of three G-quartet planes. Movement of cations between binding sites through a G-quartet plane is thus energetically more demanding than from binding site into bulk solution due to the rigidity of quadruplex core.

The present data established peculiarities of cation-binding sites in the major forms of [d(TG_3_T)]_4_ and [d(3′TG5′-5′G_2_T3′)]_4_ quadruplexes involving 5′-3′ and 5′-5′ sugar-phosphate backbone suggesting a possible role of 5′-5′ inversion of polarity sites in post-SELEX modifications of quadruplex forming aptamers (7). For example, docking studies concerning interaction between the HIV protein gp120 and an anti-HIV G-quadruplex tetramolecular aptamer (35) suggested a fundamental role for a bond between a phosphate backbone and a conserved lysine (R190) in the protein hypervariable V3 loop. The introduction of a more flexible 5′-5′ inversion of polarity site at this step, instead of a usual 5′-3′ sugar-phosphate backbone, could improve the target-aptamer interaction. The validity of this approach is under examination in our laboratories.

## CONCLUSIONS

Solution state NMR was used to get deeper insight into the role of directionality of oligonucleotide strands on G-quadruplex structural heterogeneity, localization of cation-binding sites, their occupancy and the dynamics of cation movement. A comparison of the behavior of the parent [d(TG_3_T)]_4_ with modified [d(3′TG5′-5′G_2_T3′)]_4_ and [d(5′TG3′-3′G_2_T5′)]_4_ quadruplexes confirms the better aptitude of a 5′-5′ inversion of polarity site in stabilization in comparison to a 3′-3′, because only in the first case, a stable quadruplex structure could be observed at lower concentration of ^15^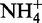 ions. The experimental data undoubtedly demonstrate that the inter-quartet cation-binding sites involving a 5′-5′ and 3′-3′ inversion of polarity sites exhibit lower occupancy in comparison to the natural 5′-3′ sugar-phosphate backbone. The comparison of the rate constants for ^15^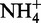 ion movements from G-quadruplex into bulk solution for investigated tetrameric quadruplex structures revealed slower cation movements at the 5′-end of the quadruplex. Furthermore, the cation movement through an all-*syn* G-quartet is slower in comparison to the movement through an all-*anti* G-quartet. No cation movements between the two binding sites of quadruplex structures consisting of three G-quartets have been observed. In general, it can be expected that cation movements in G-quadruplex structures with two binding sites are mainly between each binding site and bulk solution.

## SUPPLEMENTARY DATA

Supplementary Data are available at NAR Online: Supplementary Figures 1–7.

## FUNDING

The Slovenian research agency [ARRS, P1-0242 and J1-4020]; EU FP7 projects with acronyms EAST-NMR [228461]; Bio-NMR [261863]; COST MP0802; Ministero dell'Istruzione, Università e Ricerca (MIUR). Funding for open access charge: Slovenian Research Agency [ARRS] and Università degli Studi di Napoli Federico II Dipartimento di Chimica delle Sostanze Naturali.

*Conflict of interest statement*. None declared.

## Supplementary Material

Supplementary Data

## References

[1] Burge S, Parkinson GN, Hazel P, Todd AK, Neidle S (2006). Quadruplex DNA: sequence, topology and structure. Nucleic Acids Res..

[2] Phan AT, Kuryavyi V, Patel DJ (2006). DNA architecture: from G to Z. Curr. Opin. Struct. Biol..

[3] Keniry MA (2001). Quadruplex structures in nucleic acids. Biopolymers.

[4] Davis JT (2004). G-quartets 40 years later: from 5′-GMP to molecular biology and supramolecular chemistry. Angew. Chem. Int. Edit..

[5] Neidle S (2009). The structures of quadruplex nucleic acids and their drug complexes. Curr. Opin. Struct. Biol..

[6] Patel DJ, Phan AT, Kuryavyi V (2007). Human telomere, oncogenic promoter and 5′-UTR G-quadruplexes: diverse higher order DNA and RNA targets for cancer therapeutics. Nucleic Acids Res..

[7] Gatto B, Palumbo M, Sissi C (2009). Nucleic acid aptamers based on the G-quadruplex structure: therapeutic and diagnostic potential. Curr. Med. Chem..

[8] Bouchard PR, Hutabarat RM, Thompson KM (2010). Discovery and development of therapeutic aptamers. Annu. Rev. Pharmacol. Toxicol..

[9] Ng EW, Shima DT, Calias P, Cunningham ET, Guyer DR, Adamis AP (2006). Pegaptanib, a targeted anti-VEGF aptamer for ocular vascular disease. Nat. Rev. Drug Discov..

[10] Martino L, Virno A, Randazzo A, Virgilio A, Esposito V, Giancola C, Bucci M, Cirino G, Mayol L (2006). A new modified thrombin binding aptamer containing a 5′-5′ inversion of polarity site. Nucleic Acids Res..

[11] Esposito V, Galeone A, Mayol L, Randazzo A, Virgilio A, Virno A (2007). A mini-library of TBA analogues containing 3′-3′ and 5′-5′ inversion of polarity sites. Nucleosides Nucleotides Nucleic Acids.

[12] Esposito V, Virgilio A, Randazzo A, Galeone A, Mayol L (2005). A new class of DNA quadruplexes formed by oligodeoxyribonucleotides containing a 3′-3′ or 5′-5′ inversion of polarity site. Chem. Commun..

[13] Virno A, Zaccaria F, Virgilio A, Esposito V, Galeone A, Mayol L, Randazzo A (2007). Molecular modelling studies of four stranded quadruplexes containing a 3′-3′ or 5′-5′ inversion of polarity site. Nucleosides Nucleotides Nucleic Acids.

[14] Galeone A, Mayol L, Virgilio A, Virno A, Randazzo A (2008). A further contribution to the extreme variability of quadruplex structures from oligodeoxyribonucleotides containing inversion of polarity sites in the G-tract. Mol. Biosyst..

[15] Esposito V, Virgilio A, Pepe A, Oliviero G, Mayol L, Galeone A (2009). Effects of the introduction of inversion of polarity sites in the quadruplex forming oligonucleotide TGGGT. Bioorgan. Med. Chem..

[16] Esposito V, Oliviero G, Pepe A, Virgilio A, Galeone A (2008). Studies on the influence of inversion of polarity sites on the dG residues glycosidic conformation in quadruplex structures. Nucleic Acids Symp. Ser..

[17] Hud NV, Plavec J, Neidle S, Balasubramanian S (2006). Quadruplex Nucleic Acids.

[18] Podbevsek P, Hud NV, Plavec J (2007). NMR evaluation of ammonium ion movement within a unimolecular G-quadruplex in solution. Nucleic Acids Res..

[19] Sket P, Crnugelj M, Plavec J (2005). Identification of mixed di-cation forms of G-quadruplex in solution. Nucleic Acids Res..

[20] Podbevsek P, Sket P, Plavec J (2007). NMR study of ammonium ion binding to d[G_3_T_4_G_4_]_2_ and d[G_4_(T_4_G_4_)_3_] G-quadruplexes. Nucleosides Nucleotides Nucleic Acids.

[21] Zavasnik J, Podbevsek P, Plavec J (2011). Observation of water molecules within the bimolecular d(G_3_CT_4_G_3_C)_2_ G-quadruplex. Biochemistry.

[22] Hud NV, Schultze P, Sklenar V, Feigon J (1999). Binding sites and dynamics of ammonium ions in a telomere repeat DNA quadruplex. J. Mol. Biol..

[23] Sket P, Plavec J (2007). Not all G-quadruplexes exhibit ion-channel-like properties: NMR study of ammonium ion (non)movement within the d(G_3_T_4_G_4_)_2_ quadruplex. J. Am. Chem. Soc..

[24] Podbevsek P, Sket P, Plavec J (2008). Stacking and not solely topology of T-3 loops controls rigidity and ammonium ion movement within d(G_4_T_3_G_4_)_2_ G-quadruplex. J. Am. Chem. Soc..

[25] Akhshi P, Mosey NJ, Wu G (2012). Free-energy landscapes of ion movement through a G-quadruplex DNA channel. Angew. Chem. Int. Edit..

[26] Trajkovski M, Sket P, Plavec J (2009). Cation localization and movement within DNA thrombin binding aptamer in solution. Org. Biomol. Chem..

[27] Sket P, Plavec J (2010). Tetramolecular DNA quadruplexes in solution: insights into structural diversity and cation movement. J. Am. Chem. Soc..

[28] Hwang TL, Shaka AJ (1995). Water suppression that works—excitation sculpting using arbitrary wave-forms and pulsed-field gradients. J. Magn. Reson. Ser. A.

[29] Montelione GT, Wagner G (1989). 2d chemical-exchange NMR-spectroscopy by proton-detected heteronuclear correlation. J. Am. Chem. Soc..

[30] Sket P, Crnugelj M, Kozminski W, Plavec J (2004). ^15^NH_4_^+^ ion movement inside d(G_4_T_4_G_4_)_2_ G-quadruplex is accelerated in the presence of smaller Na^+^ ions. Org. Biomol. Chem..

[31] Virgilio A, Esposito V, Randazzo A, Mayol L, Galeone A (2005). 8-methyl-2'-deoxyguanosine incorporation into parallel DNA quadruplex structures. Nucleic Acids Res..

[32] Gros J, Rosu F, Amrane S, De Cian A, Gabelica V, Lacroix L, Mergny J-L (2007). Guanines are a quartet's best friend: impact of base substitutions on the kinetics and stability of tetramolecular quadruplexes. Nucleic Acids Res..

[33] Phong LTT, Virgilio A, Esposito V, Citarella G, Mergny J-L, Galeone A (2011). Effects of 8-methylguanine on structure, stability and kinetics of formation of tetramolecular quadruplexes. Biochimie.

[34] Cang X, Sponer J, Cheatham TE (2011). Explaining the varied glycosidic conformational, G-tract length and sequence preferences for anti-parallel G-quadruplexes. Nucleic Acids Res..

[35] Oliviero G, Amato J, Borbone N, D'Errico S, Galeone A, Mayol L, Haider S, Olubiyi O, Hoorelbeke B, Balzarini J (2010). Tetra-end-linked oligonucleotides forming DNA G-quadruplexes: a new class of aptamers showing anti-HIV activity. Chem. Commun..

