# Supplementary material for: Strand directionality affects cation binding and movement within tetramolecular G-quadruplexes
Source: Nucleic Acids Res. 2012 Sep 12;40(21):11047–57. doi: 10.1093/nar/gks851 (PMC3510487; doi:10.1093/nar/gks851)
Supplement: Supplementary Data [file supp_40_21_11047__index.html]

Strand directionality affects cation binding and movement within tetramolecular G-quadruplexes — Strand directionality affects cation binding and movement within tetramolecular G-quadruplexes — Supplementary Data 

# Strand directionality affects cation binding and movement within tetramolecular G-quadruplexes

## Supplementary Data

files

**Files in this Data Supplement:**

- Supplementary Data - pdf file
