# Supplementary material for: Strand directionality affects cation binding and movement within tetramolecular G-quadruplexes
Source: Nucleic Acids Res. 2012 Sep 12;40(21):11047–57. doi: 10.1093/nar/gks851 (PMC3510487; doi:10.1093/nar/gks851)
Supplement: Supplementary Data [file supp_gks851_nar-01826-f-2012-File009.pdf]

Supplementary material

Strand directionality affects cation binding and movement within Tetramolecular G-Quadruplexes

Primož Šket[1,2], Antonella Virgilio[3], Veronica Esposito[3], Aldo Galeone[3,*] and Janez Plavec[1,2,4,*]

[1] Slovenian NMR Center, National Institute of Chemistry, Hajdrihova 19, SI-1000 Ljubljana, Slovenia

[2] EN-FIST Center of Excellence, Dunajska 156, SI-1000 Ljubljana, Slovenia

[3] Dipartimento di Chimica delle Sostanze Naturali, Università degli Studi di Napoli Federico II, via D. Montesano 49, 80131 Napoli, Italy

[4] Faculty of Chemistry and Chemical Technology, University of Ljubljana, Askerceva cesta 5, SI-1000 Ljubljana, Slovenia

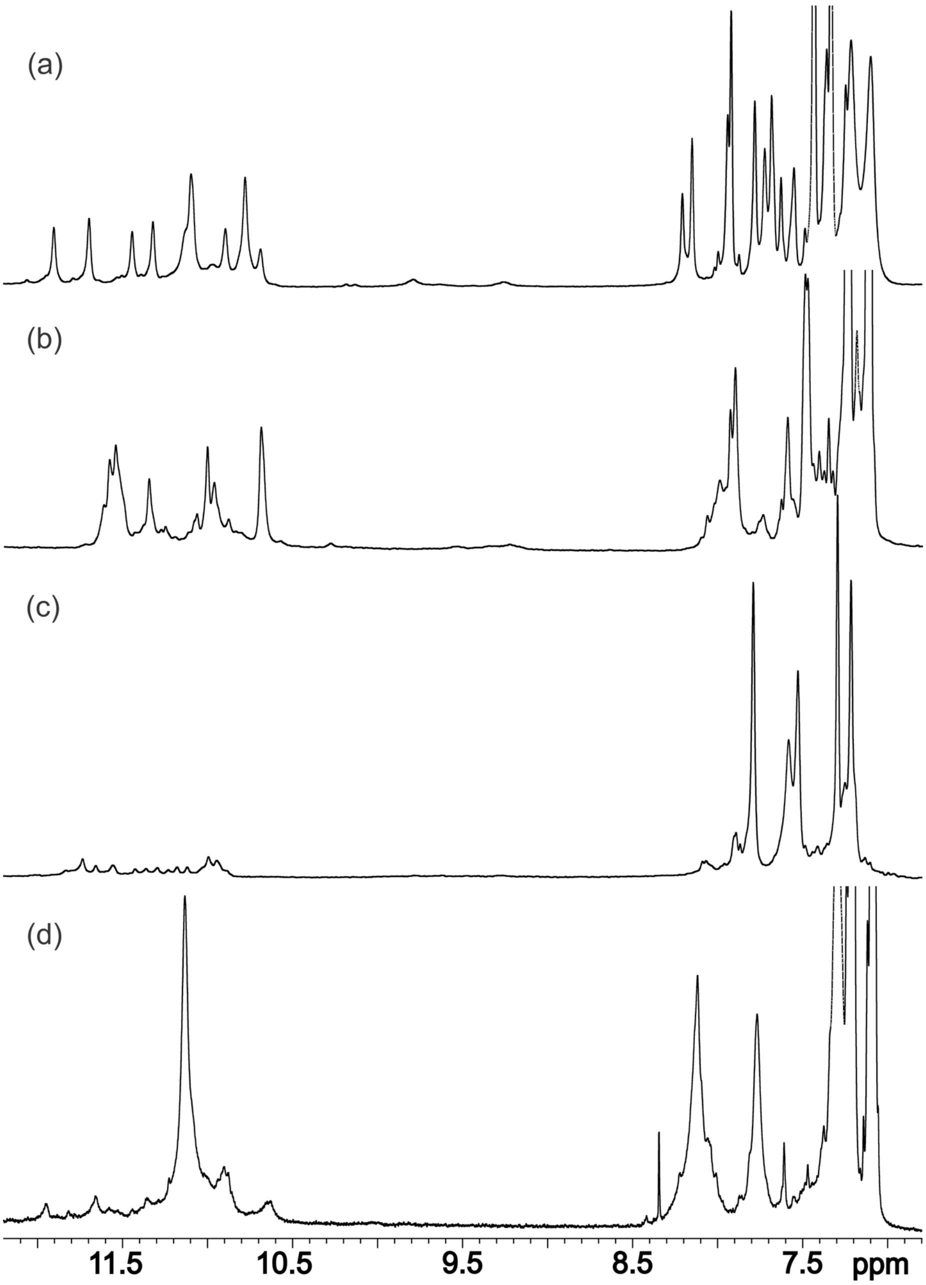

Figure S1: Imino and aromatic regions of ^{1}H NMR spectra of G-quadruplexes formed by d(TG$_3$T) (a), d(3'TG5'-5'G$_2$T3') (b), d(3'T-5'-5'G$_3$T3') (c) and d(5'TG3'-3'G$_2$T5') (d) in the presence of 10 mM concentration of $^{15}NH_4Cl$ at 0 °C in 10% ^2H_2O.

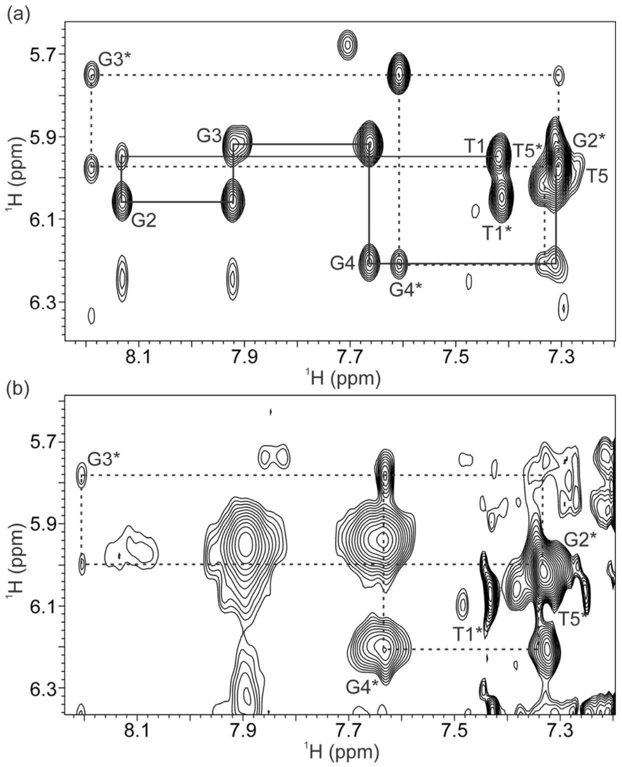

Figure S2: Aromatic-anomeric regions of NOESY spectra of oligonucleotide d(TG_3T) in the presence of 10 mM (a) and 80 mM (b) of $^{15}NH_4^+$ ions at 0 °C in 10% ^2H_2O. Mixing time was 150 ms. Sequential aromatic-H1’ connectivities in (a) for major form are labeled by solid lines, while dotted lines represent aromatic-H1’ sequential connectivities for the minor form.

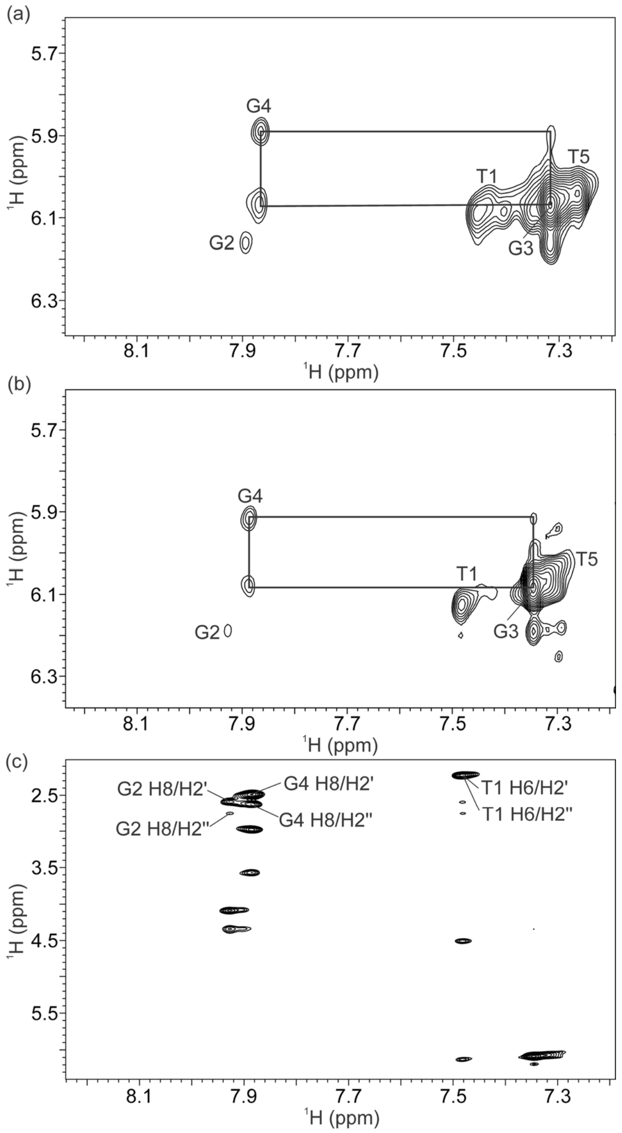

Figure S3: Aromatic-anomeric regions of NOESY spectra of oligonucleotide d(3'TG5'-5'G_2T3') in the presence of 10 mM (a) and 80 mM (b) of $^{15}NH_4^+$ ions at 0 °C in 10% ^2H_2O. Aromatic-H2′/H2″ region of NOESY spectrum of oligonucleotide d(3'TG5'-5'G_2T3') in the presence of 80 mM of $^{15}NH_4^+$ ions at 0 °C in 10% ^2H_2O (c). Mixing time was 80 ms in (a) and 150 ms in (b-c). Sequential *syn-anti* connectivities in (a-b) are labelled by solid lines.

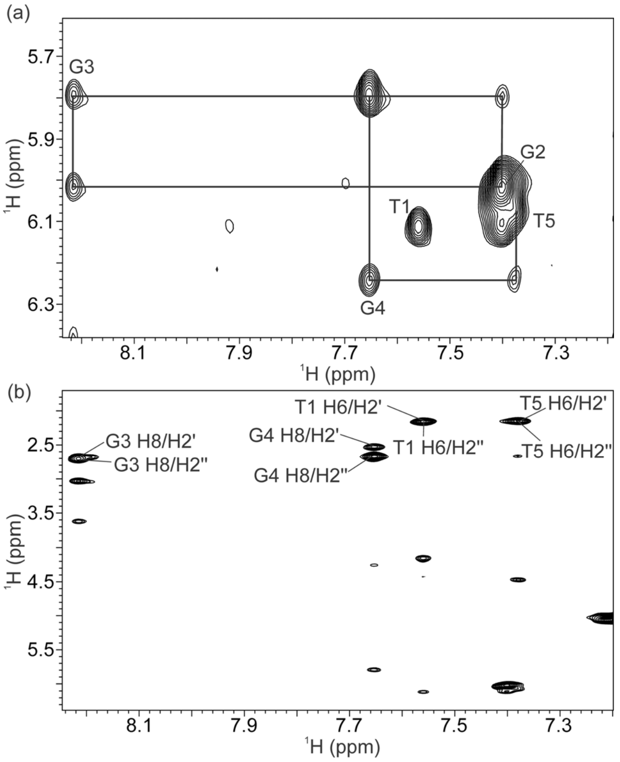

Figure S4: Aromatic-anomeric (a) and aromatic-H2′/H2″ (b) regions of NOESY spectrum of oligonucleotide d(3'T-5'-5'G_3T3') in the presence of 80 mM of $^{15}NH_4^+$ ions at 0 °C in 10% ^2H_2O. Mixing time was 150 ms. Sequential aromatic-H1' connectivities in (a) are labeled by solid lines.

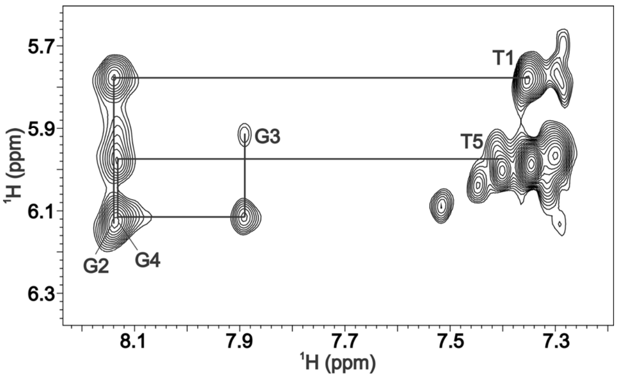

Figure S5: Aromatic-anomeric region of NOESY spectrum of oligonucleotide d(5'TG3'-3'G_2T5') in the presence of 80 mM of $^{15}NH_4^+$ ions at 0 °C in 10% ^2H_2O. Mixing time was 150 ms. Sequential aromatic-H1' connectivities are labeled by solid lines.

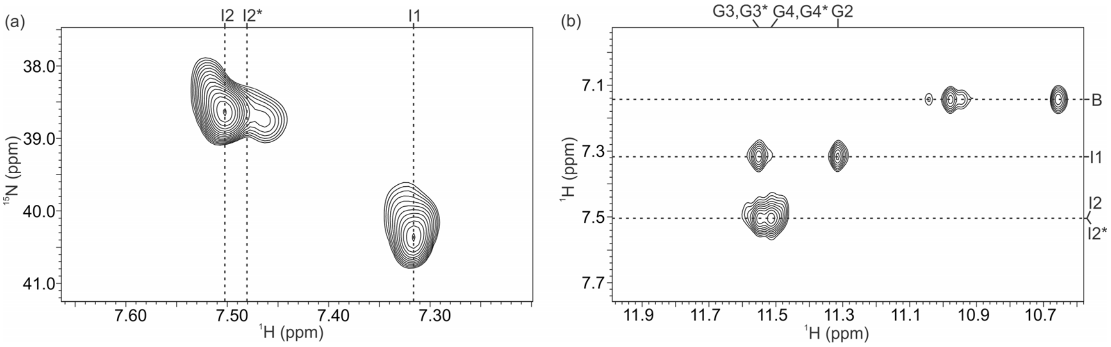

Figure S6: ^{15}N-^{1}H HSQC (a) and NOESY spectra (τ_m = 80 ms) (b) of G-quadruplex adopted by d(3'TG5'-5'G_2T3') in the presence of 10 mM $^{15}NH_4^+$ ions at 0 °C. NOESY spectrum in (b) shows cross-peaks between the bound $^{15}NH_4^+$ ions and the nearby imino protons. The label B indicates $^{15}NH_4^+$ ions in bulk solution.

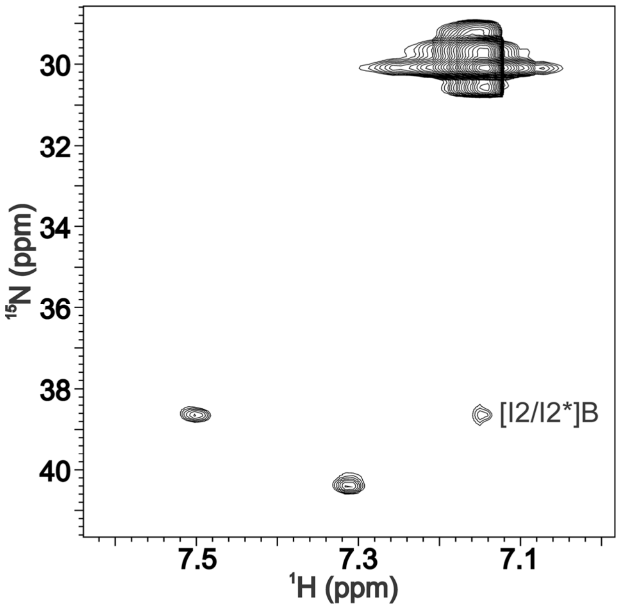

Figure S7: 2D ^{15}N-^{1}H NzExHSQC spectrum of d(3'TG5'-5'G_2T3'$)_4$ quadruplex in the presence of 80 mM $^{15}NH_4^+$ at 0 °C in 10% ^2H_2O at mixing time of 300 ms. The cross-peak label consists of two parts, where the part in brackets indicates binding sites within G-quadruplex. The first part of a label indicates the initial position, while the second part corresponds to a final site of cation movement. Only cross-peak corresponding to cation exchange is marked. Unlabeled cross-peaks correspond to $^{15}NH_4^+$ ions at different binding sites inside G-quadruplex and bulk solution. The label B indicates $^{15}NH_4^+$ ions in bulk solution.
